# Impact of COVID-19 on Lifestyle, Personal Attitudes, and Mental Health Among Korean Medical Students: Network Analysis of Associated Patterns

**DOI:** 10.3389/fpsyt.2021.702092

**Published:** 2021-08-18

**Authors:** Je-Yeon Yun, Ju Whi Kim, Sun Jung Myung, Hyun Bae Yoon, Sang Hui Moon, Hyunjin Ryu, Jae-Joon Yim

**Affiliations:** ^1^Seoul National University Hospital, Seoul, South Korea; ^2^Yeongeon Student Support Center, Seoul National University College of Medicine, Seoul, South Korea; ^3^Office of Medical Education, Seoul National University College of Medicine, Seoul, South Korea; ^4^Department of Internal Medicine, Seoul National University College of Medicine, Seoul, South Korea

**Keywords:** COVID-19, medical students, mental health, directed acyclic graph, intra-individual covariance network, lifestyle changes, fear of infection

## Abstract

The current COVID-19 pandemic have affected our daily lifestyle, pressed us with fear of infection, and thereby changed life satisfaction and mental health. The current study investigated influencing cascade of changes during the COVID-19 among the lifestyle, personal attitudes, and life (dis)satisfaction for medical students, using network-based approaches. This cross-sectional survey used self-reports of 454 medical students during June and July of 2020. Depressive mood, anxiety, and intention to drop out of school were observed in 11.9, 18.5, and 38.3% of medical students, respectively. Directed acyclic graph that estimated directional propagation of the COVID-19 in medical students' daily lives initiated from the perception of unexpected event, propagated to nervous and stressed feeling, trouble relaxing, feeling like a failure, and were followed by trouble concentrating, feeling loss of control for situation, and fear of infecting colleagues. These six features were also principal mediators within the intra-individual covariance networks comprised of changed lifestyle, personal attitude, and mental health at COVID-19 pandemic. Psychosocial supports targeting nervousness, trouble relaxing and concentrating, fear of spreading infection to colleagues, feelings of a failure or loss of situational control are required for better mental health of medical students during the COVID-19 pandemic.

## Introduction

In Republic of Korea, after the exponential increase of COVID-19 confirmed cases comprised of the multiple regional clusters including Daegu and Gyeongbuk area during January and February of 2020 (1). When this outbreak occurred, the Korean Centers for Disease Control and Prevention (KCDC) instantly dispatched public health doctors to the frontline of pandemic to enable the screening tests on all suspected COVID-19 patients, to conduct quicker quarantine of confirmed-positive patients, and to provide necessary treatment (2). During this COVID-19 pandemic, the physical and psychological burdens, as well as stresses, have been higher among medical staff members at the forefront of treating patients with COVID-19, compared with the general population (3, 4). Medical students are currently experiencing lifestyle changes similar to those of the general public, and are undergoing training as medical professionals, with the aim of preparation for future medical practice (5). For example, as a response to the sustained COVID-19 pandemic during 2020, medical schools in Republic of Korea also changed every classes to an online format from first-year to fourth-year courses except the clinical clerkship, clinical skills training, and basic laboratory classes such as anatomy lab sessions (6).

The possible after-effects of the COVID-19 pandemic include a considerably greater incidence of depressive mood and anxiety among college students after the initial pandemic wave (7). In a recent study, a significant level of psychological distress was observed among medical students in Japan who were subjected to home quarantine restrictions; greater distress was associated with reduced self-esteem and enhanced self-efficacy (8). In addition, >20% of medical students who had been quarantined in the Hubei Province of China reported moderate or severe levels of depressive mood (23.3%), anxiety (41.9%), and stress (20.9%) (9). Among medical students in the United Kingdom, considerable proportions have experienced presenteeism (40%) and reported anxiety (37.2%) and depression (46.5%) that affect life satisfaction (10). Thus, there is a need for timely assessment of interacting patterns among pandemic-related stressors [e.g., potential for transmitting COVID-19 to their families (11) and living in locations with greater COVID-19 prevalence (12)], lifestyle changes [e.g., online classes (13, 14), year of medical school (12, 15), perceived social support (16), and spare time activities and exercise (10)], and mental health factors [e.g., perceived stress, anxiety, depressive mood, history of mental health problems (16), and availability of psychological care (17)] among college students, who might be more vulnerable to COVID-19-related distress (9).

Therefore, the current study aimed to examine the interactions among the changed lifestyle (difficulty of online class attendance and use of personal time), cognitive style (perceived threat of infection & proactive coping), mental health (perceived stress, anxiety, and depressive symptoms), and school dropout intention during the COVID-19 pandemic for medical students. In the current study, we hypothesized that the environmental changes during the COVID-19 pandemic would affect the daily routines of medical students in terms of activities such as participation in online classes (rather than on-site lectures in school) and spare time activities (i.e., those influenced by social distancing). Because of the perceived threat of COVID-19 infection, changes in behavioral (social distancing and maintaining personal hygiene) and cognitive (feeling proud of medical personnel at the frontline and a willingness of volunteer) responses were expected. In the context of these ongoing readjustments, the level of life satisfaction might be reduced, thereby leading to a cascade of perceived stress, anxiety, depressive mood, and potential school dropout.

## Materials and Methods

### Participants and Study Design

The current study was conducted for the target population of medical students from the 1st to 4th grade currently enrolled in the Seoul National University College of Medicine (SNUCM) as of June and July of 2020. When students visited the campus and attended the practicum classes or sessions of academic schedule briefing during June or July of 2020, information of the current study was provided. Students with voluntary intension of participating the study could complete anonymous responses for the self-reporting questionnaires distributed in the classroom and submit the anonymous responses upon checking out of the classroom. Exclusion criteria were (1) students who had not been actively enrolled to the SNUCM as of June and July of 2020 or (2) students who did not want to participate the current study. In total, 507 of 597 students (84.9%) responded to the questionnaire. After excluding data for 53 students with missing values, our final dataset included de-identified responses from 454 medical students at Seoul National University College of Medicine during June and July of 2020. The Institutional Review Board at Seoul National University College of Medicine approved the study, and the requirement for written informed consent was waived by the board because this constituted a minimal-risk study protocol (IRB no. 2007-140-1143).

To examine the study hypotheses, the current study used three approaches. First, personal attitudes toward the COVID-19 pandemic, as well as changes in lifestyle and life (dis)satisfaction during the pandemic, were compared among subgroups of students in different years of medical school. Second, directional propagating impacts of the pandemic on the daily lives of medical students were estimated, to derive a group-wise Bayesian network: a model of probabilistic conditional dependencies among the variables of personal attitude toward the COVID-19 pandemic, changes in lifestyle, and changes in life (dis)satisfaction, depicted as a directed acyclic graph. Finally, principal influences on daily life for medical students were deciphered using intra-individual covariance networks, where the edge weights connecting two variables within an individual are proportional to the degrees of (dis)similarities between these variables in terms of the deviation from the group-averaged values of each variable. All procedures were performed in accordance with the ethical standards of the Seoul National University College of Medicine Institutional Review Board concerning human experimentation, as well as the tenets of the Helsinki Declaration of 1975, as revised in 2013.

### Difficulty of Online Class Attendance and Use of Personal Time During COVID-19 Pandemic

For more detailed profiling of the impact of COVID-19 on the medical students' daily living, the current study gathered responses concerning difficulties in participating online classes and use of personal time during the COVID-19 pandemic (Table 1 and Supplementary Material 1). First, possible difficulties of attending online classes during the COVID-19 were examined using a question of “If you experienced difficulties due to the online class operation, which of the following did you experience?” Responders were able to choose multiple items among the options of (1) maintaining regular daily routine, (2) insufficient lecturer-students interactions and related difficulties of understanding the study contents, or (3) restricted on-site social activities. For all of these three options separately, responses were binary-transformed into “perceived difficulty” or “difficulty not perceived” prior to further analyses.

**Table 1:** Demographic and clinical characteristics: sub-grouped for grade.

**Variable**	**Grade (** ***N*** **=** **454)**								**Stats**			
	**1st (** ***N*** **=** **123)**		**2nd (** ***N*** **=** **110)**		**3rd (** ***N*** **=** **121)**		**4th (** ***N*** **=** **100)**		**χ2/F**	**df**	***P***	***Post hoc***
**Demographic**: sex, men/women	80	43	70	40	75	46	64	36	0.25	3	0.969	-
**Use of personal time during COVID-19, YES/NO**												
Sleep	63	60	60	50	82	49	46	54	11.96	3	0.008	3rd > 4th
Computer game	20	103	33	77	31	90	25	75	6.44	3	0.092	-
Reading	9	114	13	97	14	107	9	91	1.84	3	0.606	-
Study	81	42	46	64	58	63	34	66	25.23	3	<0.001	1st > 2nd = 3rd = 4th
Exercise	26	97	42	68	49	72	28	72	13.36	3	0.004	1st <2nd = 3rd
Spend time with family and friends	40	83	38	72	53	68	35	65	3.90	3	0.273	-
**Difficulty of online class attendance during COVID-19, YES/NO**												
Maintaining regular daily routine	40	83	32	78	21	100	19	81	10.45	3	0.015	1st > 3rd
Insufficient interaction for understanding	31	92	15	95	23	98	28	72	8.01	3	0.046	NS
restriction of on-site social activities	65	58	62	48	60	61	46	54	2.51	3	0.473	-
**Perceived threat of infection during COVID-19, Mean/SD, [−2** **=** **strongly disagree;** **−1** **=** **disagree; 0** **=** **neither agree nor disagree; 1** **=** **agree;**												
**2** **=** **strongly agree]**												
Fear of my getting COVID-19	**–**0.5	1.1	**–**0.5	1.2	**–**0.2	1.2	0.2	1.2	19.22	3	<0.001	1st <4th, 2nd <4th
Fear of transmitting COVID-19 to family	**–**0.2	1.3	**–**0.2	1.2	**–**0.1	1.4	0.5	1.2	20.89	3	<0.001	1st <4th, 2nd <4th, 3rd <4th
Fear of transmitting COVID-19 to colleague	**–**0.2	1.2	**–**0.2	1.2	**–**0.1	1.3	0.6	1.3	28.52	3	<0.001	1st <4th, 2nd <4th, 3rd <4th
**Proactive coping for COVID-19, Mean/SD, [−2** **=** **strongly disagree;** **−1** **=** **disagree; 0** **=** **neither agree nor disagree; 1** **=** **agree; 2** **=** **strongly agree]**												
My keeping social distance	1.1	0.7	0.9	0.9	0.6	1.1	0.9	1.0	16.16	3	0.001	1st > 3rd
My keeping personal hygiene	1.3	0.8	1.2	0.7	1.1	0.8	1.2	0.8	5.96	3	0.113	-
Feeling proud for medical staff at frontline	1.7	0.6	1.5	0.8	1.3	0.9	1.3	0.9	21.69	3	<0.001	1st > 3rd, 1st > 4th
My willing to future volunteer at frontline	0.9	0.9	1.0	0.9	0.6	1.1	0.6	1.0	11.91	3	0.008	NS
**Perceived stress: PSS total score**, Mean/SD	21.0	7.6	19.6	6.2	17.9	6.5	16.6	7.0	8.92	3	<0.001	1st > 3rd, 1st > 4th, 2nd > 4th
**Anxiety: GAD-7: total score**, Mean/SD	6.6	5.1	5.5	4.3	5.0	4.9	5.0	4.9	3.10	3	0.027	1st > 3rd
**Depressive mood: PHQ-9: total score**, Mean/SD	5.7	4.6	4.3	4.5	4.0	4.6	3.5	4.6	4.89	3	0.002	1st > 3^rd^, 1st > 4th
**School dropout intention during COVID-19**	65	58	51	59	33	88	14	86	45.34	3	<0.001	1st = 2nd > 3rd = 4th

Second, pattern of personal time use during the COVID-19 pandemic was measured by way of the single question of “In the last month, which activities did you usually do during private time when you were not involved in school classes or practice?” that allowed multiple choices for a total of six options including sleep, computer game, reading, studying, physical exercise, or spending time with family and friends. Also, for all of these six options separately, responses were binary-transformed into “doing given activity in private time” or “not doing given activity in private time” for further statistical analyses.

### Perceived Threat of Infection and Proactive Coping for COVID-19 Pandemic

**Figure 1:**
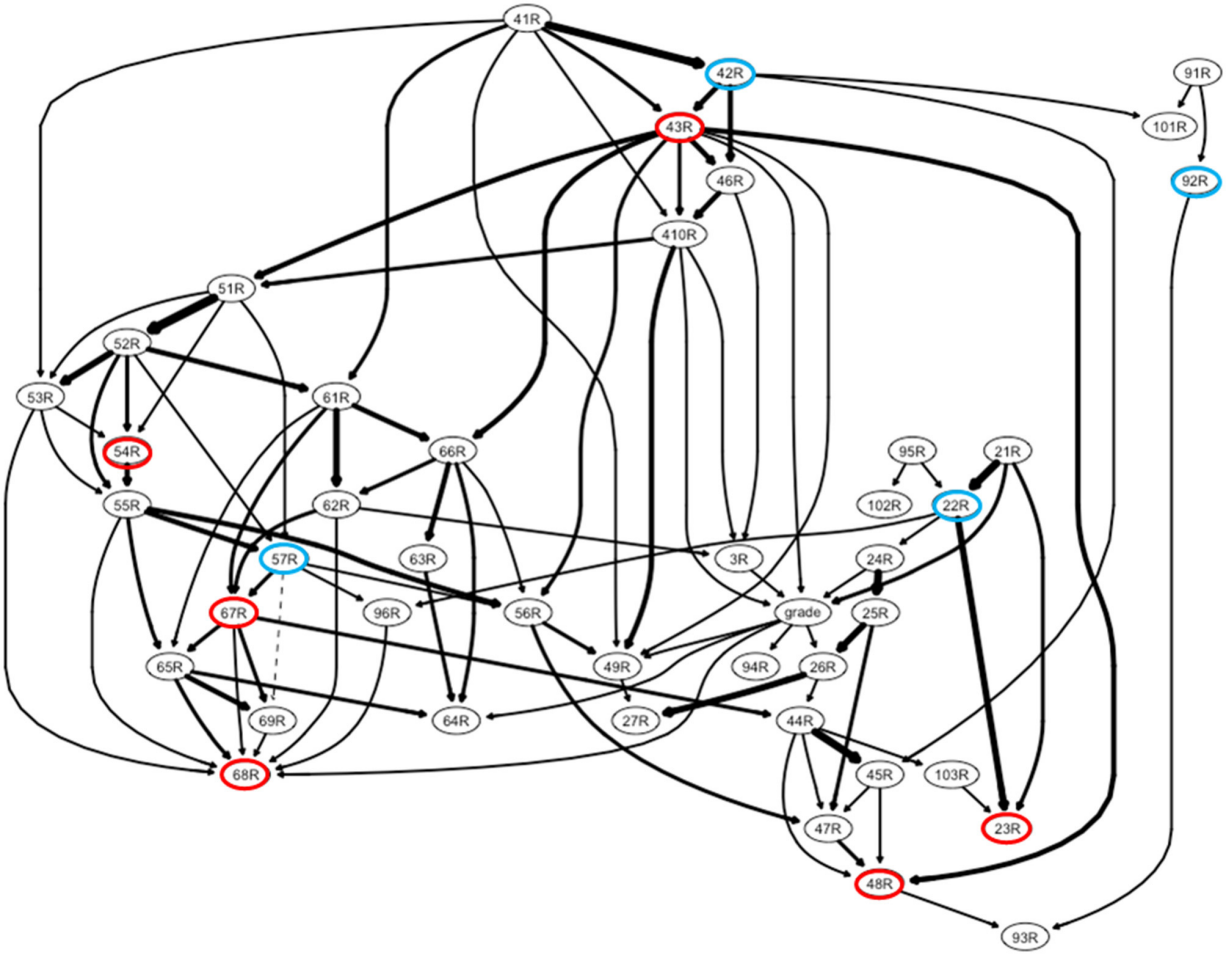
A directed acyclic graph of changes in lifestyle, personal attitudes, perceived stress, anxiety, and depressive mood among Korean medical students during the COVID-19 pandemic. The following six most influential items are marked with red circles: (1) personal attitude, fear of infecting my colleagues (R23); (2) perceived stress, feeling nervous and stressed (R43); (3) perceived stress: feeling “being on top of things” (R48); (4) anxiety, trouble relaxing (R54); (5) depressive mood, feelings of failure or that I have let myself/family members down (R67); and (6) depressive mood, trouble concentrating on things such as reading the newspaper or watching television (R68). [Personal attitude for COVID-19 pandemic] 21R = fear of my getting COVID-19; 22R = fear of my transmitting COVID-19 to family; 23R = fear of my transmitting COVID-19 to colleague; 24R = my keeping social distance; 25R = my keeping personal hygiene; 26R = feeling proud for medical staff at frontline; 27R = my willing to future volunteer at frontline/3R = intension of school dropout within recent 3 months; grade = grade as medical student/[Perceived stress] 41R = upset; 42R = unable to control; 43R = nervous or stressed; 46R = cannot cope with many things have to be done; 49R = angered for things outside of one's control; 410R = felt difficulties piled up could not be overcome; 44R = confidence for personal problems; 45R = things going one's way; 47R = control irritation; 48R = on the top of things/[Anxiety] 51R = nervous or anxious; 52R = cannot stop control worrying; 53R = worrying too much for different things; 54R = trouble relaxing; 55R = restless; 56R = easily annoyed or irritable; 57R = afraid of awful things happen/ [Depressive mood] 61R = feeling down, depressed, or hopeless; 62R = little interest or pleasure in doing things; 63R = trouble falling asleep or staying too much sleep; 64R = poor appetite or overeating; 65R = psychomotor change; 66R = tired or little energy; 67R = feel bad about oneself; 68R = trouble concentrating; 69R = idea of suicide or harming oneself/[Spare time activities in COVID-19 pandemic] 91R = sleep; 92R = computer game; 93R = reading; 94R = study; 95R = exercise; 96R = spend time with family and friends; /[Difficulties of participating in online classes in COVID-19 pandemic] 101R = maintaining regular daily routine; 102R = insufficient interaction for understanding; 103R = restriction of on-site social activities.

**Figure 2:**
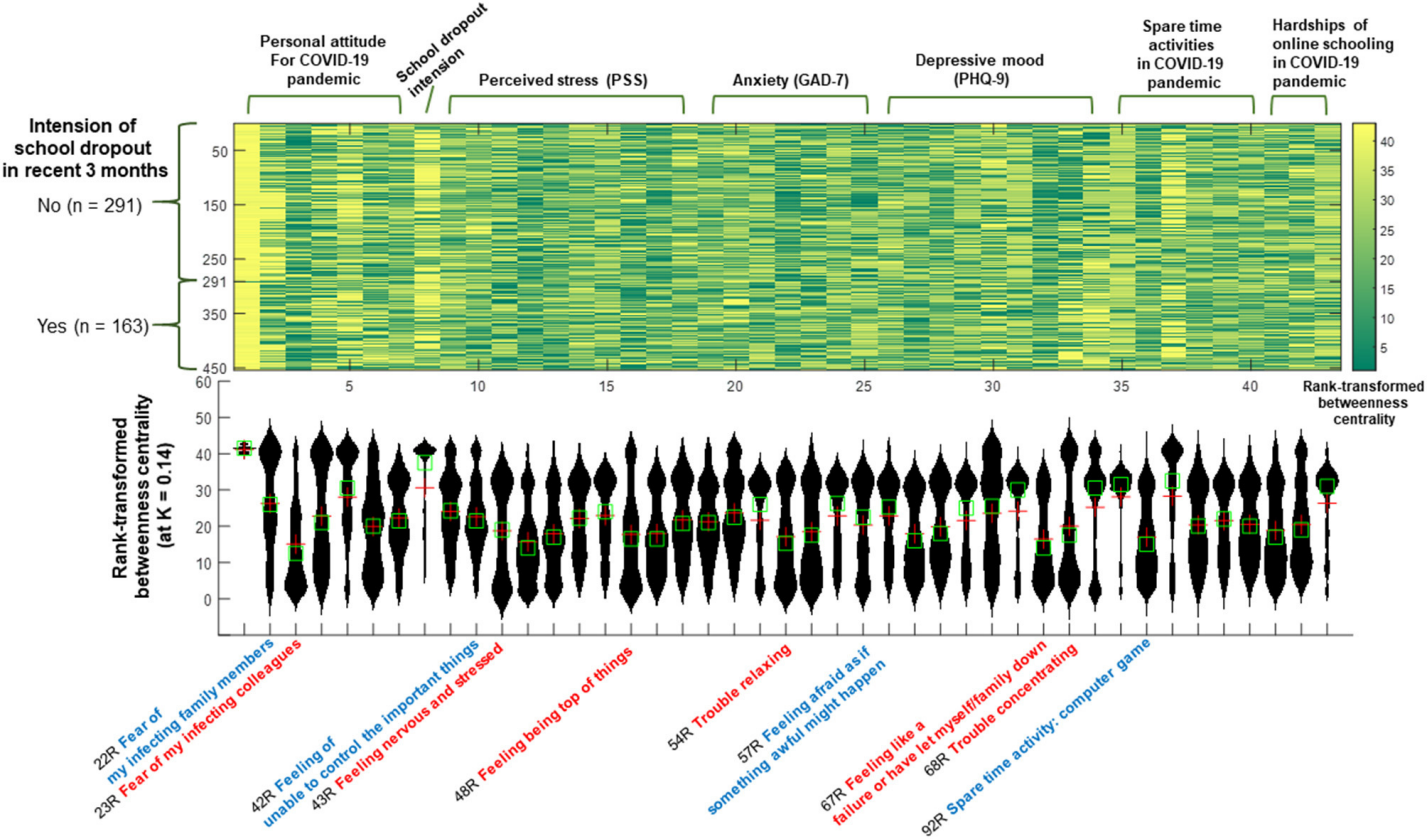
Rank-transformed betweenness centrality calculated from the intra-individual covariance networks of changes in lifestyle, personal attitudes, perceived stress, anxiety, and depressive mood among Korean medical students during the COVID-19 pandemic. In the x-axis of the violin plot (lower), the following six most influential items (hubs; top 12% for the rank-transformed betweenness centrality in ≥25% of participants (*n* = 454) at a network sparsity level of K = 0.14) are written in red: (1) personal attitude, fear of infecting my colleagues (R23); (2) perceived stress, feeling nervous and stressed (R43); (3) perceived stress: feeling “being on top of things” (R48); (4) anxiety, trouble relaxing (R54); (5) depressive mood, feelings of failure or that I have let myself/family members down (R67); and (6) depressive mood, trouble concentrating on things (R68). Items that showed significant relationships with recent intentions to drop out of school, perceived stress, or depressive mood are written in blue.

**Figure 3:**
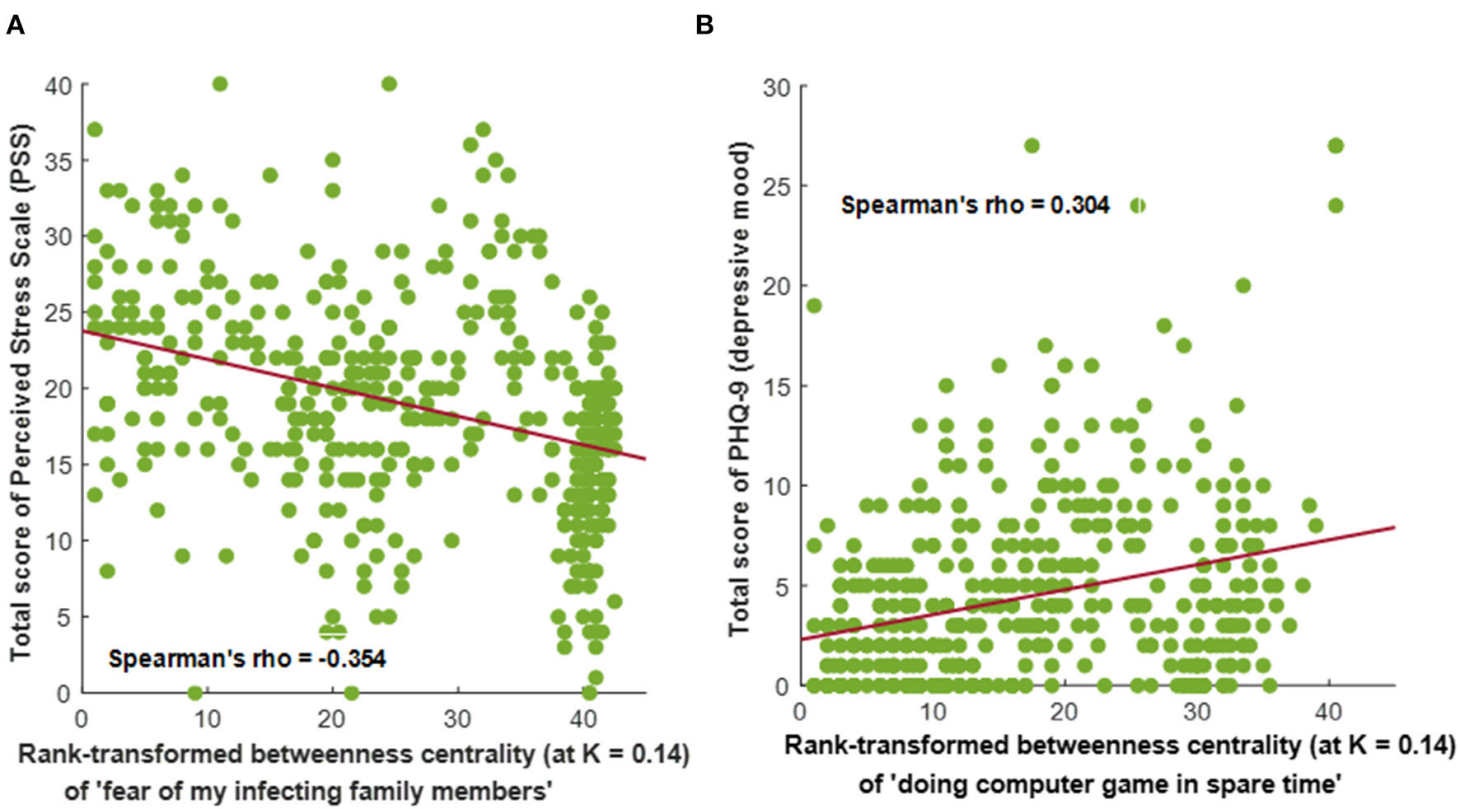
Significant correlations between the intensity of perceived stress or depressive mood vs. rank-transformed betweenness centralities of personal attitudes or changes in lifestyle during the COVID-19 pandemic among Korean medical students (*n* = 454; statistical threshold of |Spearman's rho| > 0.3 and *P* < 0.001). Values of rank-transformed betweenness centrality were calculated from the intra-individual covariance networks (at network sparsity level of K = 0.14) containing the changes in lifestyle, personal attitudes, perceived stress, anxiety, and depressive mood. **(A)** Correlations between the total score of perceived stress scale (PSS) vs. rank-transformed betweenness centrality values of “personal attitude, fear of infecting my family members” (Spearman's rho = −0.354, *P* < 0.001). **(B)** Correlations between PHQ-9 (depressive mood) total score vs. rank-transformed betweenness centrality values of “engaging in computer games in spare time” (Spearman's rho = 0.304, *P* < 0.001).

Seven questions concerning medical students' personal attitudes toward the COVID-19 pandemic were included in the current survey (Table 1 and Supplementary Material 1). First component of “proactive coping for COVID-19 as medical students and to-be medical professionals” was comprised of four items including the (1) compliance for social distancing, (2) taking care of personal hygiene, (3) feelings of pride medical staffs working at frontline, and 4) intention of future volunteering at frontline of epidemic satiations such as COVID-19 as a medical practitioner. Second component that represents “perceived threat of infection” was focused on the (1) students' fear of contracting COVID-19, and their possible roles in the transmission of COVID-19 to (2) family or (3) colleagues. Responses were acquired using a five-point Likert scale (strongly disagree, disagree, neither agree nor disagree, agree, or strongly agree), and re-coded for between-group comparison (Table 1) and network analyses (Figures 1–3).

A principal components analysis (PCA) was conducted on the seven items with orthogonal rotation (varimax). The Kaiser-Meyer-Olkin measure verified the sampling adequacy for the analysis KMO = 0.713 (fair), and all KMO values for individual items were ≥0.5, which is above the acceptable limit. Barlett's test of sphericity, χ^2^ (21) = 1,235.02, *P* < 0.001, indicating that correlations between items were sufficiently large for PCA. An initial analysis was run to obtain eigenvalues for each component in the data. A total of two components had eigenvalues over Kaiser's criterion of 1 and in combination explained 68.13% of the variance. These two components of “proactive coping” and “fear of infection” had higher reliabilities as reflected in the values of Cronbach's α = 0.762 and 0.865, respectively.

### Mental Health: Perceived Stress, Anxiety, and Depressive Symptoms

First, perceived stress during the most recent 1 month was measured using the Perceived Stress Scale (PSS) (18) validated for Korean (19). Response for the items of PSS were retrieved using the 5-point Likert scale. In the current study, value of Cronbach's α for the PSS was 0.859. Second, anxiety during the most recent 2 weeks was evaluated using the Generalized Anxiety Disorder-7 (GAD-7) (20) validated for Korean (21). Third, depressive symptoms were measured using the Patient Health Questionnaire-9 (PHQ-9) (22, 23) validated for Korean (24). Responses to each question in the GAD-7 and PHQ-9 were acquired using a four-point Likert scale. Cut-off scores of moderate depressive mood and moderate anxiety applied in the current study were PHQ-9 total score ≥10 and GAD-7 total score ≥10, respectively, as found in validation studies for Korean population (21, 24, 25). In the current study, Cronbach's α values of 0.922 and 0.859 demonstrated higher reliabilities of GAD-7 and PHQ-9, respectively.

### School Dropout Intention During the COVID-19 Pandemic

School dropout intention (26–28) during the COVID-19 pandemic was asked by way of the single question of “Have you ever considered quitting your studies in the past 3 months (=since the start of current semester (March of 2020) as of June 2020)?” Responders could choose either “yes” or “no.”

### Network Analysis: Directed Acyclic Graph

The directional propagation of the COVID-19 pandemic in medical students' daily lives, observed by means of 43 variables, were estimated as a Bayesian network using the R package named *Bnlearn* (https://www.bnlearn.com/). The 43 variables included personal attitude toward COVID-19 (seven variables of item 02), school dropout intention in the most recent 3 months (item 03), spare time activities during the COVID-19 pandemic (six variables of item 09), difficulties participating in online classes during the COVID-19 pandemic (three variables of item 10), perceived stress (10 variables of item 04; PSS), anxiety (seven variables of item 05; GAD-7), and depressive mood (nine variables of item 06; PHQ-9).

First, an optimal network structure for a bootstrapped sample [from the original dataset (*n* = 454)] was estimated using a score-based heuristic local search method, known as the hill-climbing algorithm (29, 30). After the global probability distribution (=factorization of the joint probability distribution) of the network had been determined, the parameters of local probability distributions for each node (conditional on the learned network structure) were estimated. Second, a subset of edges crucial for explaining the given sample were selected based on their higher goodness-of-fit score (e.g., Bayesian Information Criterion) (29, 30). These procedures were repeated 10,000 times, and the most consistent network edges in terms of presence and directionality were selected for the final averaged version of the directed acyclic graph. The directed acyclic graph defined probabilistic dependencies (directional edges) based on the Markov property of Bayesian networks (=direct dependence of each node only on their parental nodes) among the variables (nodes) (Figure 1) (30).

### Network Analysis: Intra-Individual Covariance Network and Graph Theory Approach

Intra-individual covariance depicts inter-item similarities and differences within each individual to determine the variance from the group-averaged value of each item. In the current study, intra-individual covariance between two different items was defined using the following formula: $\frac{1}{e^{{\left(\frac{\left(x_{A}-M_{A}\right)}{SD_{A}}-\;\frac{\left(x_{B}{-M}_{B}\right)}{SD_{B}}\right)}^{2}}}$. Thus, the intra-individual covariance value could be distributed between 0 and 1, where higher values indicate greater similarity in degrees of variance [= differences between raw values (X_A_ and X_B_) and group-averaged values (M_A_ and M_B_, *n* = 454) divided by the group-level standard deviation of each item (SD_A_ and SD_B_, *n* = 454)] between the two items of A and B within an individual (31, 32). By calculating these intra-individual covariance values among the 43 variables described above within each individual, intra-individual covariance networks were constructed for each individual (*n* = 454).

To uncover the principal influences on medical students' daily lives during the COVID-19 pandemic among these 43 variables, the current study applied the graph theory approach to these intra-individual covariance networks. First, network connectedness, small-worldness (σ, degree of balance between the overall network integration vs. network segregation into distinctive subgroups), and modularity (Q, heuristically estimated degree for a network to be subdivided into clearly delineated and non-overlapping subgroups) were derived using the network density range of K = 0.05–0.20 (with intervals of 0.1; when K = 0.05, only the top 5% largest values of intra-individual covariance survived as edges comprising an intra-individual covariance network). Second, a local network metric, known as betweenness centrality values (variable with higher betweenness centrality might be a “shortcut” among a larger number of variables that showed similar degrees of variance from group-averages within an individual), was estimated at the most sparse level of network density (K) that satisfies (1) network connectedness (>80% of items connected to each other, because they have similar degrees of variance from the group-averaged values of each variable), (2) small-world organization (σ > 1), and (3) modularity (Q > 0.3) for > 95% of participants (*n* = 454). These values were rank-transformed within each individual. All graph theory processing was conducted using the Brain Connectivity Toolbox (33).

### Statistical Analyses

According to the year of medical school, between-group comparisons of sex, school dropout intention in the most recent 3 months, spare time activities, and difficulties in online class participation were conducted using the chi-squared test of homogeneity. Concerning personal attitudes toward the COVID-19 pandemic, the Kruskal–Wallis test was applied. Total scores of PSS, GAD-7, and PHQ-9 were compared between groups using one-way analysis of variance (ANOVA). Thresholds of statistical significance were set at *P* < 0.05/3 (=number of domains including personal attitudes, changed lifestyle, and life (dis)satisfaction) = 0.017 (for main analyses) and *P* < 0.05/6 (=number of between-group comparisons) = 0.008 (for *post-hoc* analyses), respectively.

## Results

### Demographic and Clinical Characteristics

In total, 507 of 597 students (84.9%), higher percentage of response than other recent studies for medical students (34) or public health doctors (35) during COVID-19 pandemic, responded to the questionnaire. After excluding data for 53 students with missing values, our final dataset included de-identified responses from 454 medical students (123 in the first year of medical school, 110 in the second year, 121 in the third year, and 100 in the fourth year) at Seoul National University College of Medicine during June and July of 2020. Participant ages ranged from 20 to 33 years (mean age, 19.1 ± 9.0 years), and participants included 289 men (63.7%) and 165 women (36.3%). Table 1 describes between-group comparisons of (1) personal attitude toward the pandemic; (2) difficulties in online class participation during the pandemic, as well as spare time activities; (3) intensity of perceived stress-anxiety-depressive mood and school dropout intention in the most recent 3 months. Regarding personal attitudes toward the pandemic, stronger fear of contracting COVID-19 and transferring the infection to their family members or colleagues were reported by fourth-year medical students (slightly worried), compared with other medical students (not very worried; all *P* < 0.008). In addition, the first-year medical students felt greater pride for medical staff members working at the COVID-19 frontline, compared with third- or fourth-year medical students, and maintained better social distancing, compared with third-year medical students (all *P* < 0.008).

Conversely, the percentage of respondents who reported difficulty in the maintenance of a regular daily routine was higher among first-year medical students (32.5%)—who had been enrolled in online classes—than among third-year medical students (17.4%)—who were engaged in on-site hospital training. During their spare time, first-year medical students were more likely to study (65.9%) and less likely to exercise (21.1%), compared with medical students at other points in the program (41.7% for study and 40.0% for exercise). Furthermore, the mean intensity of perceived stress (total score of PSS) and depressive mood (total score of PHQ-9) were higher among first-year medical students (21.0 ± 7.6 for perceived stress and 5.7 ± 4.6 for depressive mood) than among third-year medical students (for perceived stress) and fourth-year medical students (for both perceived stress and depressive mood; all *P* < 0.008). Furthermore, moderate depressive mood (PHQ-9 total score ≥ 10) or anxiety (GAD-7 total score ≥ 10) were found in 11.9% (*n* = 54) or 18.5% (*n* = 84) of the participants, respectively. Finally, school dropout intention in the most recent 3 months (*P* < 0.001 for main analysis) was higher in first- and second-year medical students (49.8%) than in third- and fourth-year medical students (21.3%).

### Propagated Impacts of COVID-19 in Daily Lives of Medical Students: Directed Acyclic Graph

Using item-level responses for the whole dataset (*n* = 454), a group-wise directed acyclic graph was established to uncover the propagating patterns among the following items: (1) personal attitude toward pandemic; (2) changes in lifestyle (difficulties in online class participation during the pandemic and spare time activities during the pandemic); and (3) changes in life (dis)satisfaction (perceived stress, anxiety, depressive mood, and school dropout intention in the most recent 3 months). As shown in Figure 1, the results indicated that medical students' distress during the COVID-19 pandemic was initiated by the perception of unexpected events related to pandemic (41R). Moreover, it extended to the fear of transferring COVID-19 to colleagues (23R), perceived stress [nervous and stressed feelings (43R) and feeling a loss of situational control (48R)], anxiety [trouble relaxing (54R)] and depressive mood [feelings of failure (67R) and trouble concentrating (68R); all items listed above are marked as red circles in Figure 1].

### Principal Influences on Medical Students' Daily Lives During the COVID-19 Pandemic: Graph Theory Approach for the Intra-Individual Covariance Network

The principal influences on personal attitudes, changes in lifestyle, and changes in life (dis)satisfaction during the COVID-19 pandemic were identified using rank-transformed betweenness centrality values (Figure 2), estimated from the intra-individual covariance networks (containing items also in the directed acyclic network; Figure 1) at the sparsity level of K = 0.14 (in which the top 14% of edges with higher covariance values survived) that satisfied the following criteria for > 95% of participants (*n* = 454): (1) network connectedness (>80% of items connected to each other), (2) small-worldness (sigma > 1), and (3) modularity (Q > 0.3).

Accordingly, the following six items were ranked as top 10% items (=rank-transformed betweenness centrality ≤ 4) for more than 40% of participants: (1) fear of transferring COVID-19 to colleagues (23R; personal attitude), (2) nervous and stressed feelings (43R; perceived stress), (3) feeling a loss of situational control (48R; perceived stress), (4) trouble relaxing (54R; anxiety), feelings of failure (67R; depressive mood), and trouble concentrating (68R; depressive mood). These items were selected as principal influences (marked with red-rimmed circles in Figure 1; names written in red color at the bottom of Figure 2).

### Differential Patterns of Connectedness According to Life (dis) Satisfaction During the COVID-19 Pandemic

Correlation analyses between the severity of perceived stress (total score of PSS), anxiety (total score of GAD-7), and depressive mood (total score of PHQ-9) vs. rank-transformed centrality derived from the intra-individual covariance networks uncovered associations between higher rank of betweenness centrality for the “fear of transmitting COVID-19 to family members (22R)” and higher perceived stress (Spearman's rho = −0.354, *P* < 0.001; Figure 3A). In contrast, a higher rank of betweenness centrality for “engaging in computer games in spare time (92R)” was associated with lower depressive mood (Spearman's rho = 0.304, *P* < 0.001; Figure 3B).

## Discussion

### Study Summary

To our knowledge, this study is the first to decipher the influencing cascade of changed lifestyle (difficulty of online class attendance and use of personal time), cognitive style (perceived threat of infection & proactive coping), mental health (perceived stress, anxiety, and depressive symptoms), and school dropout intention during the COVID-19 pandemic for medical students, by means of network-based approaches. For reducing the possible transmission of COVID-19 by way of on-site interpersonal interactions, medical schools in Republic of Korea also changed most of the classes to an online format from first-year to fourth-year courses (6). For basic laboratory classes such as anatomy lab sessions, students were equipped with personal protective equipment students and were divided into smaller groups to reduce the spread of possible infections (6). After the partial loosening of social distancing as of May of 2020 by announcement of government, core clinical clerkship programs were re-opened at training hospitals and conducted in compliance with preventive guideline for COVID-19 pandemic (6).

In the current study, depressive mood, anxiety, and intention of school dropout were observed in 11.9, 18.5, and 38.3% of medical students, respectively. These tendencies were more prominent among junior medical students. The current results are not higher than the prevalence of clinically relevant depressive symptoms measured during the pre-pandemic era for medical students worldwide (27–28%) (36, 37) and in South Korea (10.9–23%) (38–40), who had already been reporting higher levels of depressive mood, anxiety, and psychological distress, compared with the general population (41). However, because the profile of psychological responses to stressful situations can vary among individuals, network analyses were conducted to uncover the possible directional cascade of psychological symptom progression and core influencing components. A directed acyclic graph began from the perception of unexpected events; then transitioned to nervous and stressed feelings, trouble relaxing, and feelings of failure; and finally progressed to trouble concentrating, feeling a loss of situational control, and fear of infecting colleagues. These six features were also highly ranked for betweenness centrality in the intra-individual covariance networks. Of note, perceived stress showed a negative association with rank-transformed betweenness centrality of “fear of infecting my family members (Spearman's rho = −0.354)”; in contrast, a higher rank of “engaging in computer games in spare time” for betweenness centrality was associated with lower depressive mood (PHQ-9 total score; Spearman's rho = 0.304) (all *P* < 0.001).

### Influencing Patterns Among Perceived Stress, Personal Attitudes, and Changes in Lifestyle

In the current study, perceived stress in response to the COVID-19 pandemic began from the surprise concerning the unexpected occurrence of the COVID-19 pandemic. After this feeling of surprise transitioned into nervousness and distress, medical students experienced feelings of anxiety (“on edge”), irritability, and fatigue. When medical students who had been using spare time for sleeping felt that they were unable to control the important things in their lives, they experienced difficulty in maintaining a regular daily routine during social distancing. This is consistent with other studies, which showed that college students during the COVID-19 pandemic experienced distress when adjusting to new academic activities and changes in sleeping pattern. Their social isolation and “all-or-none” cognitive style could lead to worsened mental health and life satisfaction (42, 43). Furthermore, when they had not been able to cope with all the tasks they had to complete and felt that difficulties were becoming so extensive that they could not be managed, medical students with little interest or pleasure in doing things sometimes considered taking a leave of absence from school. Timely provision of academic mentoring and networking, as well as psychological care for possible depressive moods, might be crucial in minimizing unintended leaves of absence from school by medical students during the COVID-19 pandemic (44–47).

In contrast, the level of confidence that they are in control of a situation and aware of changes, and whether they used spare time for computer games, might influence the use of spare time to read books. Notably, reading books has been widely used to aid in coping with sustained adaptation distress among veterans (48), burnout among oncologists (49), and physical illnesses [e.g., hemodialysis (50)]. Furthermore, medical students who have been angered because of things outside of their control, but also felt pride in seeing medical staff members at the COVID-19 frontline, reported a willingness to volunteer to work as a medical professional at the frontline of future epidemic situations. As a possible proactive coping mechanism, some of them volunteered as peer-tutors (51).

### Influencing Patterns Among Anxiety, Personal Attitudes, and Changes in Lifestyle

To control the amplification of anxiety among medical students during the COVID-19 pandemic, capacities for voluntary relaxation and maintenance of social ties with family and friends might be helpful. The current study showed that ~18.5% of medical students reported anxiety (GAD-7 total score ≥ 10). Because they already feel distress and experience hopelessness about the increasing difficulties, initial anxiety that involves feeling nervous, anxious, or on edge might escalate. Thus, the students may be unable to stop or control worrying about various things. Because sustained worrying could lead to trouble relaxing and subsequent fear of an awful outcome, preemptive application of progressive muscle relaxation (52) or the therapeutic use of a coloring book (53) might be suggested.

Importantly, medical students who had a fear of transmitting COVID-19 to family members and a fear of an awful outcome used their spare time to see family and friends. Of note, the severity of perceived stress (= total score of PSS) was higher in medical students for whom the transformed z-score value [using the means and standard deviations of given items calculated from all participants (*n* = 454)] of fear for transmitting COVID-19 to family members was similar to most other personal attitude-changed lifestyle-life (dis)satisfaction items (i.e., higher-ranked values of betweenness centrality derived from the intra-individual covariance network; Spearman's rho = −0.354, *P* < 0.001). Because a weak sense of coherence is associated with greater risks of mood disturbance and anxiety during the COVID-19 pandemic (54), medical staff members at the COVID-19 frontline also require familial support and social connectedness to mitigate the fear of infection (55).

### Influencing Patterns Among Depressive Mood, Personal Attitudes, and Changes in Lifestyle

Lowered self-efficacy could be a principal influence on the progression of depressive symptoms and distress. In the current study, ~11.9% of medical students reported a depressive mood (PHQ-9 total score ≥ 10). Sustained surprise and uncontrollable worrying during the COVID-19 pandemic could result in feeling “down,” depressed, or hopeless. Furthermore, if medical students do not experience much interest or pleasure in their tasks and instead exhibit fear of an awful outcome, they might regard themselves as failures and have reduced self-confidence in handling personal problems. As symptoms of depressive mood and anxiety worsen, medical students complain of concentration difficulty. Altered confidence in handling personal problems would be followed by changes in capacity for controlling irritation, life contentment, and feelings of situational control and awareness of changes. To prevent the worsening of depressive moods among medical students during the COVID-19 pandemic, there is a need for balancing of a negative cognitive style and collective evaluation tendencies by means of Socratic questioning and more objective evaluations of tasks based on actual evidence (56, 57).

### Limitations

This study had some limitations. First, the current study is cross-sectional, and therefore, comparisons with pre-pandemic period per study participant were not possible. Recent studies showed an increased prevalence of psychiatric symptoms such as distress, anxiety, insomnia, and depressive mood during the COVID-19 pandemic, compared with pre-pandemic assessments (58). Throughout follow-up studies during this pandemic, the prevalence of symptoms was stable (59) or decreasing (60, 61), compared with earlier stages. Further longitudinal cohort studies (62, 63) are needed to understand the long-term after-effects of the COVID-19 pandemic on the interacting pattern among personal attitudes, changes in lifestyle, and changes in life (dis)satisfaction. Second, three items used in the current study in measurements of “difficulty of online class attendance (1 item) & use of personal time (1 item) during COVID-19 pandemic” and “school dropout intention during the COVID-19 pandemic (1 item)” were not validated in the current study. Third, the current study did not explore the possible mediation effect of socioeconomic status between the COVID-19 pandemic and its impact on medical students. Specifically, people with lower socioeconomic backgrounds could find difficulties in adjusting themselves among the changing situation of COVID-19 (64). Fourth, the directed acyclic graph applied in the current study was based on probabilistic and causal modeling, and did not consider the possibility of bidirectional interactions among variables. Future studies might be suitable for exploring such bidirectional interactions by applying newly proposed tools [e.g., interaction directed acyclic graph (65)] that have been sufficiently verified.

### Conclusions

Overall, the current study examined the influencing cascade of changes in lifestyle, personal attitudes, and life (dis)satisfaction among medical students during the COVID-19 pandemic using network-based approaches. To minimize distress propagation, timely control is necessary concerning the following principal influences: nervous and stressed feelings, trouble relaxing, feelings of failure, trouble concentrating, fear of infecting colleagues, and feeling a loss of situational control.

## Data Availability Statement

The datasets used in the current study are available from the corresponding author (Sun Jung Myung, issac73@snu.ac.kr) on reasonable request.

## Ethics Statement

The studies involving human participants were reviewed and approved by the Institutional Review Board at Seoul National University College of Medicine approved the study protocol, and the requirement for written informed consent was waived by the board because this constituted a minimal-risk study. Written informed consent for participation was not required for this study in accordance with the national legislation and the institutional requirements.

## Author Contributions

J-YY, JK, SMy, HY, SMo, HR, and J-JY conceived and designed the study idea. J-YY and MJ managed literature searches and wrote the manuscript. J-YY, JK, SMy, HY, SMo, HR, and J-JY critically reviewed the manuscript. All authors contributed to and have approved the final manuscript.

## Conflict of Interest

The authors declare that the research was conducted in the absence of any commercial or financial relationships that could be construed as a potential conflict of interest.

## Publisher's Note

All claims expressed in this article are solely those of the authors and do not necessarily represent those of their affiliated organizations, or those of the publisher, the editors and the reviewers. Any product that may be evaluated in this article, or claim that may be made by its manufacturer, is not guaranteed or endorsed by the publisher.
