# Supplementary material for: Impact of COVID-19 on Lifestyle, Personal Attitudes, and Mental Health Among Korean Medical Students: Network Analysis of Associated Patterns
Source: Front Psychiatry. 2021 Aug 18;12:702092. doi: 10.3389/fpsyt.2021.702092 (PMC8416342; doi:10.3389/fpsyt.2021.702092)
Supplement: Supplementary file 1 [file Table_1.DOCX]

**Supplemental Information**

Impact of COVID-19 on lifestyle, personal attitudes, and mental health among medical students: network analysis of associated patterns

**S1. Survey of distress among medical students in the context of the COVID-19 pandemic**

1. Sex: male female / Age: ( ) years

2. [COVID-19] Please answer the following questions.

| No. | item | Strongly disagree | Disagree | Neither agree nor disagree | Agree | Strongly agree |
| --- | --- | --- | --- | --- | --- | --- |
| 1 | I am worried that wearing a mask and keeping the physical distance between people alone will not protect me from COVID-19 infection during on-site classes and practice. |  |  |  |  |  |
| 2 | I am worried that my family will be infected with COVID-19 because of me. |  |  |  |  |  |
| 3 | I am worried that my hospital and university members will be infected with COVID-19 because of me. |  |  |  |  |  |
| 4 | As a medical student aiming to become a medical professional, I am keeping social distancing (by reducing the visit to public spaces and participation of social gatherings, among others). |  |  |  |  |  |
| 5 | As a medical student aiming to become a medical professional, I take good care of personal hygiene. |  |  |  |  |  |
| 6 | I am proud to see the medical staff working in the medical field to respond to COVID-19. |  |  |  |  |  |
| 7 | I am willing to volunteer to work in the medical field in an epidemic situation such as COVID-19 as a medical practitioner in the future. |  |  |  |  |  |

3. Have you ever wanted to quit your studies in the last 3 months?: yes no

4. [Perceived Stress Scale] The questions in this scale ask you about your feelings and thoughts during the last month. In each case, you will be asked to indicate by circling how often you felt or thought a certain way.

0 = Never 1 = Almost Never 2 = Sometimes 3 = Fairly Often 4 = Very Often

| No. | item | 0 | 1 | 2 | 3 | 4 |
| --- | --- | --- | --- | --- | --- | --- |
| 1 | In the last month, how often have you been upset because of something that happened unexpectedly? |  |  |  |  |  |
| 2 | In the last month, how often have you felt that you were unable to control the important things in your life? |  |  |  |  |  |
| 3 | In the last month, how often have you felt nervous and “stressed”? |  |  |  |  |  |
| 4 | In the last month, how often have you felt confident about your ability to handle your personal problems? |  |  |  |  |  |
| 5 | In the last month, how often have you felt that things were going your way? |  |  |  |  |  |
| 6 | In the last month, how often have you found that you could not cope with all the things that you had to do? |  |  |  |  |  |
| 7 | In the last month, how often have you been able to control irritations in your life? |  |  |  |  |  |
| 8 | In the last month, how often have you felt that you were on top of things? |  |  |  |  |  |
| 9 | In the last month, how often have you been angered because of things that were outside of your control? |  |  |  |  |  |
| 10 | In the last month, how often have you felt difficulties were piling up so high that you could not overcome them? |  |  |  |  |  |

5. [General Anxiety Disorder-7] Over the last two weeks, how often have you been bothered
by any of the following problems?

| No. | items | Strongly disagree | Several days | More than half the days | Nearly every day |
| --- | --- | --- | --- | --- | --- |
| 1 | Feeling nervous, anxious or on edge? |  |  |  |  |
| 2 | Not being able to stop or control worrying? |  |  |  |  |
| 3 | Worrying too much about different things? |  |  |  |  |
| 4 | Trouble relaxing? |  |  |  |  |
| 5 | Being so restless that it is hard to sit still? |  |  |  |  |
| 6 | Becoming easily annoyed or irritable? |  |  |  |  |
| 7 | Feeling afraid as if something awful might happen? |  |  |  |  |

6. [Patient Health Questionnaire-9] Over the last two weeks, how often have you been bothered by any of the following problems?

| No. | items | Strongly disagree | Several days | More than half the days | Nearly every day |
| --- | --- | --- | --- | --- | --- |
| 1 | Feeling down, depressed, or hopeless? |  |  |  |  |
| 2 | Little interest or pleasure in doing things? |  |  |  |  |
| 3 | Trouble falling or staying asleep, or sleeping too much? |  |  |  |  |
| 4 | Poor appetite or overeating? |  |  |  |  |
| 5 | Moving or speaking so slowly that other people could have noticed? Or the opposite - being so fidgety or restless that you have been moving around a lot more than usual? |  |  |  |  |
| 6 | Feeling tired or having little energy? |  |  |  |  |
| 7 | Feeling bad about yourself - or that you are a failure or have let yourself or your family down? |  |  |  |  |
| 8 | Trouble concentrating on things, such as reading the newspaper or watching television? |  |  |  |  |
| 9 | Thoughts that you would be better off dead, or of hurting yourself in some way? |  |  |  |  |

6-1) Did you have trouble working, doing housework, and getting along with people because of these problems?

| No problem at all | It was a little hard | It was very difficult | It was very hard |
| --- | --- | --- | --- |
|  |  |  |  |

9. In the last month, which activities did you usually do in private time when you were not involved in school classes or practice? (Multiple responses available)

(1) sleep

(2) computer game

(3) reading

(4) studying

(5) exercise

(6) spend time with family and friends

10. If you experienced difficulties due to non-face-to-face class operation, which of the following inconveniences did you experience?

(1) Maintaining regular daily routine

(2) Insufficient interaction for understanding

(3) Restriction of on-site social activities
